# Supplementary material for: Critical Assessment of MetaProteome Investigation (CAMPI): a multi-laboratory comparison of established workflows
Source: Nat Commun. 2021 Dec 15;12:7305. doi: 10.1038/s41467-021-27542-8 (PMC8674281; doi:10.1038/s41467-021-27542-8)
Supplement: Supplementary file 3 — Description of Additional Supplementary Files [file 41467_2021_27542_MOESM3_ESM.pdf]

Description of Additional Supplementary Files

File Name: Supplementary Data 1
Description: This file contains an overview of the LC-MS/MS experiments and all their metadata for S01-S12 and F01-F09 used in this study.

File Name: Supplementary Data 2
Description: Summarizes the LC-MS/MS experiments, contains search metadata and highlights the samples selected for all further analyses.

File Name: Supplementary Data 3
Description: An analysis of search spaces concerning the four protein sequence databases used (SIHUMIx reference database, SIHUMIx metagenomic database, fecal reference database and fecal metagenomic database). This analysis was moved entirely to the supplementary material during revision.

File Name: Supplementary Data 4
Description: This is the result of a search for contradictory functional annotation in protein groups and subgroups and between the two grouping approaches used. It shows the largest proteins for groups/PAPPSO, groups/MPA, subgroups/PAPPSO and subgroups/MPA. A contradictory annotation was apparent for groups, but not for subgroups.

File Name: Supplementary Data 5
Description: This is the full data analysis for protein grouping methods that resulted in Supplementary Data 4 (which is contained in the first sheet of this Excel file). Further sheets in this file contain the calculations for the number of groups/subgroups and the distribution of spectral/protein count for groups/subgroups for the eight different methods listed in the summary, and are deprecated prophane output data files (full files found in the GitHub repository, protein grouping).

File Name: Supplementary Data 6
Description: Contains further metadata of the comparison between protein grouping methods. The script to generate these numbers are found in the GitHub repository (protein grouping). The source data for the table metadata were the same prophane output data files used for Supplementary Data 5.

File Name: Supplementary Data 7
Description: The result file of an advanced Unipept search for SIHUMIx. Taxonomic distributions for samples and relevant taxonomic genera.

File Name: Supplementary Data 8
Description: The result file of an advanced Unipept search with missed cleavage handling (MCH) for SIHUMIx. Full taxonomic annotation for peptides.

File Name: Supplementary Data 9
Description: The result file of an advanced Unipept search with missed cleavage handling (MCH) for fecal sample. Full taxonomic annotation for peptides.

File Name: Supplementary Data 10
Description: Full Prophane summary.txt file for SIHUMIx/reference database. Contains the subgroups, taxonomic and functional annotation and spectral count quantitation for all LC-MS/MS experiments.

File Name: Supplementary Data 11
Description: Full Prophane summary.txt file for fecal sample/metagenome. Contains the subgroups, taxonomic and functional annotation and spectral count quantitation for all LC-MS/MS experiments.

File Name: Supplementary Data 12
Description: Full Prophane summary.txt file for SIHUMIx/metagenome. Contains the subgroups, taxonomic and functional annotation and spectral count quantitation for all LC-MS/MS experiments.

File Name: Supplementary Data 13
Description: Taxonomic annotation of protein subgroups SIHUMIx/reference database.

File Name: Supplementary Data 14
Description: Taxonomic annotation of protein subgroups fecal sample/metagenome.

File Name: Supplementary Data 15
Description: Peptide matrix for SIHUMIx/reference database. Contains all found peptides and indicates their presence/absence (0/1) in each sample.

File Name: Supplementary Data 16
Description: Peptide matrix for fecal sample/metagenome. Contains all found peptides and indicates their presence/absence (0/1) in each sample.
