# Supplementary material for: Critical Assessment of MetaProteome Investigation (CAMPI): a multi-laboratory comparison of established workflows
Source: Nat Commun. 2021 Dec 15;12:7305. doi: 10.1038/s41467-021-27542-8 (PMC8674281; doi:10.1038/s41467-021-27542-8)
Supplement: Supplementary file 6 — Supplementary Dataset 3 [file 41467_2021_27542_MOESM6_ESM.html]

Search space size analysis


# Comparison of the search space size¶

Here, we will compare the number of possible in silico digested peptides between the databases used in the metaproteomics contest paper.

## SIHUMIx: UniProt Reference proteomes (DB1) vs Metagenome (DB2)¶

In [10]:

```
SIHUMI_DB1 = set()
SIHUMI_DB2 = set()

with open("./170508_SIHUMI_n8_species_cRAP.cpdt", "r") as in_f:
    for line in in_f:
        if line.startswith(" PEPTIDE"):
            SIHUMI_DB1.add(line.split(" ")[2].split(":")[0].strip())

with open("./SIHUMI_2_MG/SIHUMI_MG_Db_crap_.cpdt", "r") as in_f:
    for line in in_f:
        if line.startswith(" PEPTIDE"):
            SIHUMI_DB2.add(line.split(" ")[2].split(":")[0].strip())
```

In [15]:

```
print("SIHUMIx - DB1:", len(SIHUMI_DB1))
print("SIHUMIx - DB2:", len(SIHUMI_DB2))
print("SIHUMIx - Intersection:", len(SIHUMI_DB1.intersection(SIHUMI_DB2)))
print("SIHUMIx - only in DB1:", len(SIHUMI_DB1 - SIHUMI_DB2))
print("SIHUMIx - only in DB2:", len(SIHUMI_DB2 - SIHUMI_DB1))
```

```
SIHUMIx - DB1: 1701122
SIHUMIx - DB2: 1068489
SIHUMIx - Intersection: 799698
SIHUMIx - only in DB1: 901424
SIHUMIx - only in DB2: 268791
```

In [12]:

```
# library
import matplotlib.pyplot as plt
from matplotlib_venn import venn2
 
venn2(subsets = [SIHUMI_DB1, SIHUMI_DB2], set_labels = ('SIHUMIx_DB1', 'SIHUMIx_DB2'))
plt.show()
```

# Gut: IGC (DB1) vs Metagenome (DB2)¶

In [6]:

```
Gut_DB1 = set()
Gut_DB2 = set()

with open("./GUT_1_IGC/gut_1_igc_.cpdt", "r") as in_f:
    for line in in_f:
        if line.startswith(" PEPTIDE"):
            Gut_DB1.add(line.split(" ")[2].split(":")[0].strip())

with open("./GUT_2_Metagenomics/Gut_2_MG.cpdt", "r") as in_f:
    for line in in_f:
        if line.startswith(" PEPTIDE"):
            Gut_DB2.add(line.split(" ")[2].split(":")[0].strip())
```

In [13]:

```
print("Gut - DB1:", len(Gut_DB1))
print("Gut - DB2:", len(Gut_DB2))
print("Gut - Intersection:", len(Gut_DB1.intersection(Gut_DB2)))
print("Gut - only in DB1:", len(Gut_DB1 - Gut_DB2))
print("Gut - only in DB2:", len(Gut_DB2 - Gut_DB1))
```

```
Gut - DB1: 329131464
Gut - DB2: 20083298
Gut - Intersection: 17548410
Gut - only in DB1: 311583054
Gut - only in DB2: 2534888
```

In [9]:

```
# library
import matplotlib.pyplot as plt
from matplotlib_venn import venn2
 
venn2(subsets = [Gut_DB1, Gut_DB2], set_labels = ('Gut_DB1', 'Gut_DB2'))
plt.show()
```

In [ ]:

```

```
