# Supplementary material for: Critical Assessment of MetaProteome Investigation (CAMPI): a multi-laboratory comparison of established workflows
Source: Nat Commun. 2021 Dec 15;12:7305. doi: 10.1038/s41467-021-27542-8 (PMC8674281; doi:10.1038/s41467-021-27542-8)
Supplement: Supplementary file 20 — Reporting Summary [file 41467_2021_27542_MOESM20_ESM.pdf]

nature portfolio

Corresponding author(s): Lennart Martens

Last updated by author(s): Oct 18, 2021

Reporting Summary

Nature Portfolio wishes to improve the reproducibility of the work that we publish. This form provides structure for consistency and transparency in reporting. For further information on Nature Portfolio policies, see our Editorial Policies and the Editorial Policy Checklist.

Statistics

For all statistical analyses, confirm that the following items are present in the figure legend, table legend, main text, or Methods section.

n/a	Confirmed	
☐	☒	The exact sample size (*n*) for each experimental group/condition, given as a discrete number and unit of measurement
☐	☒	A statement on whether measurements were taken from distinct samples or whether the same sample was measured repeatedly
☒	☐	The statistical test(s) used AND whether they are one- or two-sided<br>*Only common tests should be described solely by name; describe more complex techniques in the Methods section.*
☒	☐	A description of all covariates tested
☒	☐	A description of any assumptions or corrections, such as tests of normality and adjustment for multiple comparisons
☐	☒	A full description of the statistical parameters including central tendency (e.g. means) or other basic estimates (e.g. regression coefficient) AND variation (e.g. standard deviation) or associated estimates of uncertainty (e.g. confidence intervals)
☒	☐	For null hypothesis testing, the test statistic (e.g. F, t, r) with confidence intervals, effect sizes, degrees of freedom and P value noted<br>*Give P values as exact values whenever suitable.*
☒	☐	For Bayesian analysis, information on the choice of priors and Markov chain Monte Carlo settings
☐	☒	For hierarchical and complex designs, identification of the appropriate level for tests and full reporting of outcomes
☐	☒	Estimates of effect sizes (e.g. Cohen's *d*, Pearson's *r*), indicating how they were calculated

Our web collection on statistics for biologists contains articles on many of the points above.

Software and code

Policy information about availability of computer code

Data collection	We have used the following software packages + version number: Integrated Meta-Omics Pipeline 2, MEGAHIT 1.2.4, MetaBAT 2.12.1, MaxBin 2.2.6, DASTool 1.1.2, Prodigal 2.6.3, SearchGUI 3.3.3, PeptideShaker 1.16.23, X!Tandem ALANINE (2017.02.01), MaxQuant 1.6.5, MS-GF+ 2018.01.30, Comet 2018.01 rev. 0, MetaProteomeAnalyzer 3.4, ProteomeDiscoverer 2.2, X!TandemPipeline (PAPPSO) 0.4.3, Unipept 4.2, Unipept Desktop 1.2.1, Unipept CLI 2.2.1, MegaGO 0.5.1, Prophane 5.0, mOTU profiler 2.0, Kraken 2, bowtie 2, featureCounts 2.0.1, Mantis 1.1.1, R 4.0.2, UpSetR package 1.4.0, R prcomp package 4.0.2, corrplot R package 0.84.
Data analysis	All scripts are made available on github.com/metaproteomics/CAMPI.

For manuscripts utilizing custom algorithms or software that are central to the research but not yet described in published literature, software must be made available to editors and reviewers. We strongly encourage code deposition in a community repository (e.g. GitHub). See the Nature Portfolio guidelines for submitting code & software for further information.

Data

Policy information about availability of data

All manuscripts must include a data availability statement. This statement should provide the following information, where applicable:

- Accession codes, unique identifiers, or web links for publicly available datasets
- A description of any restrictions on data availability
- For clinical datasets or third party data, please ensure that the statement adheres to our policy

The metaproteomic data sets generated and analyzed in the current study are available via the PRIDE partner repository with the data set identifier PXD023217. Assemblies and raw metagenomic and metatranscriptomic reads are available through the European Nucleotide Archive under the study accession number PRJEB42466.

Furthermore, we used the following public databases: Human Genome version 38, IGC catalog 9.9, UniProtKB - reference proteomes SIHUMIx downloaded in July 2019, the proteomics contaminant database (cRAP) downloaded in July 2019, human UniProtKB reference proteome downloaded in September 2019, NCBI non-redundant database downloaded in September 2019, eggNOG 4.5.1, Pfam-A 32, maxikraken2 1903.

Field-specific reporting

Please select the one below that is the best fit for your research. If you are not sure, read the appropriate sections before making your selection.

☒ Life sciences ☐ Behavioural & social sciences ☐ Ecological, evolutionary & environmental sciences

For a reference copy of the document with all sections, see nature.com/documents/nr-reporting-summary-flat.pdf

Life sciences study design

All studies must disclose on these points even when the disclosure is negative.

Sample size	Two samples (SIHUMIx from UfZ Leipzig and fecal sample from University of Magdebourg) were distributed across several labs worldwide. Regarding the determination of the sample size: this is relevant when performing a clinical or biological study, in order to subset a population and adequately represent the population and draw conclusions. Here, the human gut sample came from only one individual and was used to compare metaproteomics methods from different laboratories, and not to draw any biological or clinical conclusions.
Data exclusions	No data exclusions.
Replication	Although we have some technical replicates, most of the samples were only processed once. In this manuscript this is not a problem because no biological or clinical conclusions were drawn.
Randomization	No biological or clinical studies were performed. In this manuscript, we compared two different samples from two different environments which were only sampled once and distributed to different labs to compare their metaproteomics methods. Therefore, no randomization was needed.
Blinding	No clinical or biological studies were performed. Here, we compared two different samples from two different environments which were sampled only once and distributed to different labs worldwide in order to compare metaproteomics methods. We did not compare different conditions nor draw any biological or clinical conclusions. Blinding was thus not relevant here. In fact, it was rather the opposite. In order to choose the best metaproteomics methods, laboratories needed to know which kind of samples they were dealing with.

Reporting for specific materials, systems and methods

We require information from authors about some types of materials, experimental systems and methods used in many studies. Here, indicate whether each material, system or method listed is relevant to your study. If you are not sure if a list item applies to your research, read the appropriate section before selecting a response.

Materials & experimental systems

n/a	Involved in the study	
☒	☐	Antibodies
☒	☐	Eukaryotic cell lines
☒	☐	Palaeontology and archaeology
☒	☐	Animals and other organisms
☐	☒	Human research participants
☒	☐	Clinical data
☒	☐	Dual use research of concern

Methods

n/a	Involved in the study	
☒	☐	ChIP-seq
☒	☐	Flow cytometry
☒	☐	MRI-based neuroimaging

Human research participants

Policy information about studies involving human research participants

Population characteristics	A natural human fecal microbiome sample was procured upon informed consent from a 33-year old, omnivorous, non-smoking woman.
Recruitment	One sample delivered from one co-worker at the University of Magdeburg. We did not perform comparison between i.e different conditions nor draw any biological conclusions. There was thus no bias to be introduced. Moreover, this person was selected because, being familiar with our study, she volunteered.
Ethics oversight	The Ethics Committee of the University of Magdeburg (number 99/10).

Note that full information on the approval of the study protocol must also be provided in the manuscript.
